# Computerized clinical decision system and mobile application with expert support to optimize management of vertigo in primary care: study protocol for a pragmatic cluster-randomized controlled trial

**DOI:** 10.1007/s00415-020-10078-0

**Published:** 2020-07-27

**Authors:** Filipp M. Filippopulos, Doreen Huppert, Thomas Brandt, Margit Hermann, Mareike Franz, Steffen Fleischer, Eva Grill

**Affiliations:** 1 German Center for Vertigo and Balance Disorders, LMU Klinikum, Ludwig Maximilians Universität, Marchioninistraße 15, 81377 Munich, Germany; 2 Department of Neurology, LMU Klinikum, Ludwig Maximilians Universität, Marchioninistraße 15, 81377 Munich, Germany; 3 AOK Bayern, Die Gesundheitskasse, Carl-Wery-Straße 28, 81739 Munich, Germany; 4 Kassenärztliche Vereinigung Bayerns, Referat Versorgungsinnovationen, Elsenheimerstraße 39, 80687 Munich, Germany; 5 Institute for Health and Nursing Sciences, Medical Faculty, Martin Luther University Halle-Wittenberg, Magdeburger Straße 8, 06112 Halle (Saale), Germany; 6 Institute of Medical Informatics, Biometry and Epidemiology, Ludwig Maximilians Universität, Marchioninistraße 15, 81377 Munich, Germany

**Keywords:** Vertigo, Dizziness, Primary care, Automized clinical decision system

## Abstract

Vertigo and dizziness are amongst the most common symptoms in medicine and often have a major impact on activities of daily life. Although many causes of vertigo and dizziness can easily be recognized, patients often receive inappropriate and ineffective treatment. The reasons for this are various. Because vertigo/dizziness is an interdisciplinary symptom and there is a lack of standardised diagnostic tools, it is easy to lose the overview of the possible differential diagnoses. There is evidence though, that the management of patients with vertigo/dizziness can be optimized using standardized care pathways with digital support. The present study (within the framework of “PoiSe—prevention, online feedback, and interdisciplinary therapy of acute vestibular syndromes by e-health”) aims to evaluate the implementation of a program with several interlocking components. The three main components are a computerized clinical decision system, a mobile application, a counselling and interdisciplinary educational program developed by the German Center for Vertigo and Balance Disorders (DSGZ). The study is a cluster-randomized controlled trial with a parallel-group design, as well as a detailed process evaluation. Clusters comprise of primary care physician practices in Bavaria, Germany. In the scope of the study the effectiveness, acceptability and efficiency of the intervention will be evaluated. It is anticipated that the intervention will improve the quality and efficiency of the management of dizzy patients. A higher diagnostic accuracy, optimized treatment, and disease progression monitoring is expected to improve patient-relevant outcomes and reduce health-care costs.

## Background

Vertigo and dizziness are amongst the most common key symptoms in medicine. Lifetime prevalence has been shown to lie between 20 and 30% [1, 2]. Claims data from the Bavarian Statutory Health Insurance Physicians’ database showed that over 9% of the adult population received a diagnosis of vertigo annually, most of these in primary care [3]. Disorders of the central or peripheral vestibular system may lead to severe permanent disability or to considerable restrictions of daily life [4]. Although most syndromes—as a rule—can be correctly diagnosed and managed by specialists with interdisciplinary training [5], there is still a large discrepancy between recommended and actual management of patients with vertigo [6, 7]. This might lead not only to overuse of health care resources [4, 8], but also to a prolonged clinical course and to the development of chronic complaints or somatization. Notably, primary care physicians perceive diagnosis and treatment of vertigo as complex and challenging while they feel that they risk missing life-threatening conditions such as stroke or neoplasms [7]. Also, while monitoring symptom evolution under treatment, which is of the utmost relevance for the disease management, it has been noted that the resources of a typical primary care consultation are too limited to closely monitor patients with vertigo. Likewise, the narrow view of single aspects of the underlying disorders reflects the compartmentalization of the clinical specializations (neurology, otorhinolaryngology, internal medicine, etc.) with non-uniform clinical guidelines, and insufficient interdisciplinary cooperation [9].

To overcome these problems of management, monitoring and lack of interdisciplinary consultation, various computerized solutions have been proposed. Computerized clinical decision support systems (CCDS) are technologies that aim to optimize clinical decision-making in situations of uncertainty and complexity [10]. Evidence for their effectiveness has been shown for care process parameters such as the ordering of diagnostic tests [11] or medication prescription. Evidence for their effectiveness on patient-reported outcomes, however, is still limited [12]. Secondly, various mobile solutions for the management of chronic disease and risk factors, e.g. for type 2 diabetes [13], bipolar disorders [14], headache [15] or congestive heart failure [16] that have the potential to facilitate monitoring therapy adherence and success are currently being developed. Thirdly, in remote rural areas tele-medicine applications are increasingly used to support primary care decisions with expert advice (e.g. in stroke [17] or emergency medicine [18]; for review see [19]). All these solutions are often implemented as isolated interventions without overarching concept of care and without dedicated evaluation of the implementation processes and their barriers.

We therefore hypothesized that a combination of CCDS with an easy-to use mobile application and systematic expert support would be able to improve diagnostic accuracy and outcomes of patients presenting with acute vertigo syndromes in primary care, if supported by a large network of different stakeholders.

The objective of this study is therefore to evaluate the implementation of an innovative program with several interlocking components. Specifically, we want to examine the effectiveness, acceptability and efficiency of the new program in primary care.

## Methods

The study protocol follows the Standard Protocol Items: Recommendations for International Trials (SPIRIT).

### Trial design

The present study is a cluster-randomized controlled trial with a parallel-group design (PG-CRCT), including a 6-month pilot phase. A detailed process evaluation alongside the trial will be carried out.

Clusters will be primary care practices in Bavaria, one of Germany’s 16 federal states with a population of about 13 millions. Clusters will be randomly assigned in a balanced 1:1 block design to either the intervention or the control group after inclusion into the main study. Concealed allocation of participating practices will be implemented centrally by an independent person that has no knowledge of the practice identity. The randomization sequence will be computer-generated. The size of the practice (number of patients per year) will be considered as a stratification factor. Practices with special focus on vestibular or balance disorders will be excluded.

The study will be submitted to the local ethics committee for approval and prospectively registered in the German Clinical Trials Register (DRKS) before inclusion of the first patient.

### Participants

*Clusters* Practices in Bavaria will be eligible if they are led by at least one primary care physician and have patients insured by the Allgemeine Ortskrankenkasse Bayern (AOK Bayern), one of the largest health insurance funds in Bavaria with approximately 4.5 millions insured patients. As insured patients are entitled to freely choose their physician and some patients might directly consult medical specialists, neurologists, otorhinolaryngologists and internal medicine specialists will be included in addition to family practitioners.

*Patients* Consenting patients will be eligible for inclusion if they newly consult a participating practice for acute vertigo or dizziness (ICD-10 codes (H81.1–H81.3, H81.8, H81.9, R42 with G63, E53.8, N95.1, G62, I951, H55 or F45.8), are at least 18 years old, and are mentally able to follow instructions. They must also be insured by the AOK Bayern. Patients will be excluded if they are suffering from a mental illness (e.g. dementia), or a terminal disease with a life expectancy of less than 12 months.

### Intervention

**Fig. 1:**
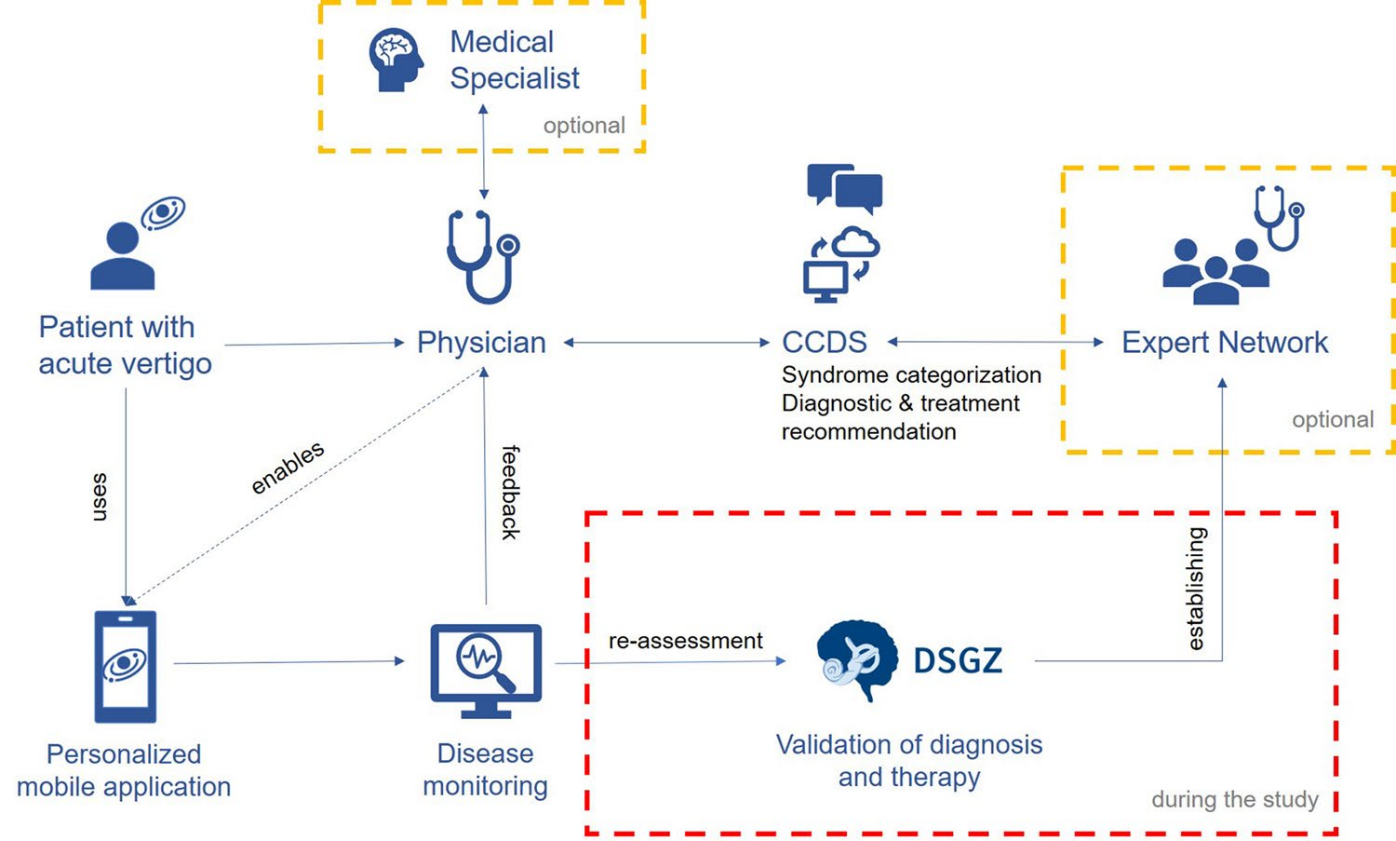
Summary of the process flow of the intervention. During the study period the German Center for Vertigo and Balance Disorders (DSGZ) will re-assess all patients for the purpose of validating the diagnosis and treatment. Further, the expert network will be established throughout the study period

The intervention consists of three basic components: a web-based platform (CCDS), a mobile application, and a structured system for continuing medical education, expert support and auditing (see Fig. 1).

Patients presenting with acute vertigo at the physician’s office will be eligible for inclusion in the study. After inclusion, the physician (family practitioner, neurologist, otorhinolaryngologist, internal specialist) will use the CCDS. The patient’s answers to a few basic questions (e.g. How long does the vertigo last?), symptoms (e.g. vomiting) and clinical findings (e.g. horizontal nystagmus) will be analysed by an AI-based algorithm. Subsequently suggestions are offered to the physician for a) a categorization of the possible underlying syndrome (e.g. peripheral vs. central vertigo), b) required diagnostic procedures, c) treatment options, and d) referral to physicians with a different medical speciality (e.g. referral from a family practitioner to a neurologist). Additionally, the physician will receive a warning message when symptoms of a severe underlying disease are noted (e.g. skew deviation in cerebral stroke).

Questionnaires and underlying decision algorithms are based on national and international guidelines and the International Classification of Vestibular Disorders (ICVD) of the Bárány Society.

After the diagnostic process has been completed, the patient is given access to a mobile application (app). This application informs the patient about the underlying disease, supports the therapeutic process and monitors the course of the disease. The app is personalized to the patient’s demographic data (age, gender, language, visual impairment) and to the diagnosis. Furthermore, patients will be instructed to fill out a vertigo/dizziness diary on a daily basis. A newly developed, AI-based algorithm then monitors the information gathered from the diary and correlates it with the individual disease. Additionally, an integrated video documentation and analysis system will analyse eye movements and gait abnormalities by the means of pattern recognition (e.g. neuronal networks, deep learning algorithms) [20–22]. It will be used to objectify the diagnosis and classify patient’s self-recorded symptoms.

In case of an unwanted outcome, e.g. signs of a somatization disorder or no visible improvement of symptoms, the app informs the patient and the physician about this development and suggests that the diagnosis and treatment be reconsidered. The physician additionally has access to an expert network for further support.

The expert network will be established throughout the study period. Physicians with special interest in vestibular disorders, taking geographic criteria into consideration, will be selected to participate in interdisciplinary professional training sessions, video-based case review sessions and consultations by phone. The aim is to create a broad network of physicians that are able to correctly diagnose and treat patients with vertigo/dizziness using an interdisciplinary approach. This is especially relevant for rural areas or regions with a sparse medical network. The expert network overall is intended to complement the digital solution.

**Fig. 2:**
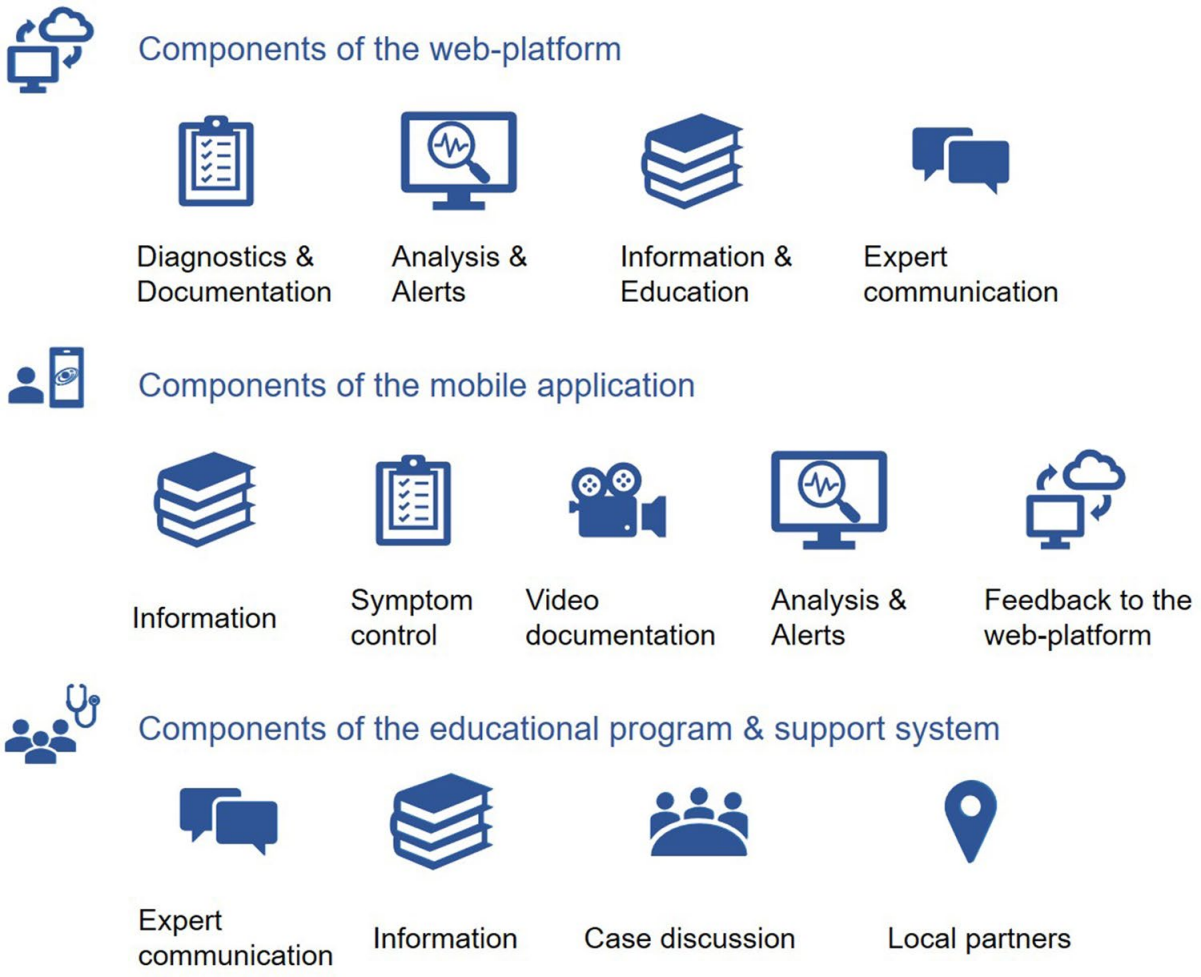
Contents of the three main components of the intervention: the web-platform with the CCDS, the mobile application, and the educational/support system

The anticipated contents of the three main components are summarized in Fig. 2. The intervention will be tested in a 6-month pilot period. Adaptations of the intervention and the contents after this time-period are possible (see below).

### Data collection procedures

Data will be collected at physician (cluster) and patient level. Patient data will be collected at inclusion and after 3 and 6 months. After three months, all patients will be invited to be re-assessed at the German Center for Vertigo and Balance Disorders (DSGZ; see Fig. 1), an outpatient tertiary university unit specialized in the diagnosis and treatment of vertigo and balance disorders. Re-assessments will be blinded to the initial diagnosis and to the study group. Data management will follow the General Data Protection Regulation.

### Measures

The outcomes were chosen to measure the effectiveness, acceptability and efficiency of the intervention at provider (cluster) and patient level.

To measure effectiveness at the provider level, the accuracy of diagnoses made by the primary care physician will be evaluated by re-assessment at the DSGZ. Primary effectiveness outcomes at the patient level will be measured using the Dizziness Handicap Inventory (DHI), a validated instrument for measuring disease-specific quality of life and participation in social life [23]. The DHI scores between 0 and 100, with higher scores indicating a decrease in quality of life. Generic quality of life will be measured using the EuroQol-5D (EQ-5D) [24].

The acceptability and feasibility of the intervention will be assessed using a mixed methods approach. Qualitative data will be ascertained by purposeful sampling through individual and group interviews [25]. Quantitative data will be gathered by questionnaires and standardized documentation of the processes throughout the entire study period (including access data generated from the web-platform and the mobile application).

Efficiency will be assessed in terms of the utilization of health care services and the respective costs. To this end, routine data provided by the health insurer AOK Bayern will be linked to individual patient data from the trial. We will concentrate on outpatient visits, hospitalizations, visits to the emergency room, and imaging, because these seem to be the major cost drivers [26].

### Process evaluation

Process evaluation will be conducted according to the guidelines of the Medical Research Council (MRC) Framework for evaluating complex interventions [27]. During an initial 6-month pilot period, the acceptance and feasibility of the intervention and its components will be evaluated using a mixed-methods approach. In the following main trial (16 months of recruitment of patients) the processes and quality of the implementation will be evaluated. We will evaluate various aspects, including the structure and performance of the included practices, implementation fidelity, satisfaction with the intervention and implementation, assessment of scalability, and documentation and assessment of unexpected effects. Auditing will include quality parameters such as study protocol adherence and number of protocol violations, data quality, and adherence to data protection protocols.

### Harms

The intervention is not expected to lead to any adverse events. Serious unexpected effects believed to be related to the intervention will be collected and recorded according to the GCP from randomization of the first patient through 90 days after the inclusion of the last patient. In this case the intervention will be discontinued, and the patient will be referred to a medical expert.

### Sample size

A difference of 6–10 points in the DHI with a standard deviation of 20 points is assumed to be a relevant clinical difference. This corresponds to an effect size of between 0.3 and 0.5 [6]. A two-sided t-test for independent groups with an effect size of 0.3, an alpha of 0.05 and beta of 0.20 (power = 80%) results in a sample size of *n* = 352 individuals. The inclusion of 10 patients per cluster was assumed with a drop-out rate of 10%, which corresponds to the drop-out rate of similar studies [28, 29]. Therefore, the inclusion of nine patients per cluster was assumed. Taking into account a conservative inter-cluster correlation coefficient (ICC) of 0.04 [30], a design effect (DE) of 1.32 was calculated (DE = 1 + (*k* – 1)  × ICC). This results in a sample size of *n* = 468 patients (234 in the intervention program and 234 in the standard care) and thus 52 clusters. At the level of the clusters, a drop-out rate of 2 clusters per study arm can be expected [31]. To further compensate for possible variations in patient recruitment of the clusters, two additional clusters per study arm were considered. Thus, a total of 60 clusters with 600 patients was calculated.

The additional 6-month pilot phase will include 10 clusters with an expected recruitment of 10 patients per cluster (total *n* = 100 patients; 50 for each study arm). The aim of the pilot phase is to gather information to optimize the study processes (recruitment, randomisation, data gathering, etc.) and to adapt/improve the CCDS and mobile application. The measured outcomes of the pilot phase will not be included in the evaluation and statistical analysis of the main study.

## Discussion

We expect that the intervention will improve the quality and effectiveness of the management of patients presenting with acute vertigo or dizziness.

The intervention is expected to optimize the diagnostic decision process, thus, leading to a timely and more effective therapy and a relevant improvement of patient-relevant outcomes. Also, the mobile application will facilitate treatment adherence and symptom management.

Potential limitations that have to be considered include issues of acceptability and usability of the mobile application and interoperability of the web solution. Special needs of persons with disabilities and older persons will have to be considered. This increased diagnostic accuracy and optimized treatment has the potential to improve long term outcomes and reduce health-care costs by reducing the number of diagnostic procedures, the number of consultations, and the treatment costs.
